# MTA-cooperative PRMT5 inhibitors from cofactor-directed DNA-encoded library screens

**DOI:** 10.1073/pnas.2425052122

**Published:** 2025-05-16

**Authors:** Jan Andersson, Sanne Cowland, Mikkel Vestergaard, Yajing Yang, Siyuan Liu, Xie Fang, Susmith Mukund, Sudipa Ghimire-Rijal, Chris Carter, Grace Chung, Tomas Jacso, Ian Sarvary, Paul E. Hughes, Alex Gouliaev, Marc Payton, Brian Belmontes, Sean Caenepeel, Thomas Franch, Sanne Glad, Birgitte Husemoen, Søren Jensby Nielsen

**Affiliations:** ^a^Amgen Research, Copenhagen DK-2100, Denmark; ^b^Amgen Research, Thousand Oaks, CA 91320; ^c^Amgen Research, South San Francisco, CA 94080

**Keywords:** DNA-encoded library screens, PRMT5, cancer

## Abstract

Methylthioadenosine phosphorylase (*MTAP*) gene deletions are frequent in human cancers. Loss of *MTAP* leads to significantly increased cellular levels of methylthioadenosine (MTA), a cellular metabolite and specific inhibitor of the cell-essential enzyme Protein Arginine Methyltransferase-5 (PRMT5). Using a cofactor-directed screening strategy and DNA-encoded libraries, we identify a class of PRMT5 inhibitors that cooperatively inhibit PRMT5 in the presence of MTA. An optimized inhibitor, AM-9934, selectively inhibits PRMT5 in *MTAP*-deleted cells and in transplanted tumors while sparing *MTAP*-expressing counterparts, leading to specific suppression of viability in *MTAP*-deleted cells. Structural studies show that AM-9934 occupies the arginine substrate pocket of MTA-bound PRMT5. This study introduces a broadly applicable method for directed DNA-encoded library screening toward a desired mechanistic outcome and highlights MTA-selective PRMT5 inhibition as an attractive therapeutic strategy with a potentially broad therapeutic index in patients with *MTAP*-deleted cancers.

## Significance

The methylthioadenosine phosphorylase (*MTAP*) gene is recurrently lost in human cancers. A methylthioadenosine (MTA)-directed DNA-encoded library screening strategy was employed to identify MTA-cooperative PRMT5 binders, leading to development of AM-9934. AM-9934 inhibits PRMT5 only in the presence of MTA and selectively impairs proliferation of *MTAP* -deleted cancer cells and growth of *MTAP^−^*^/^*^−^* tumors while sparing *MTAP*^+/+^ cells.

Genomic alterations in cancers create dependencies that can be exploited for precision therapy. Gene amplification or mutational activation of oncogenes has been successfully targeted by inhibitors directly addressing the oncogenic lesion, as exemplified by Herceptin in Her2-amplified breast cancer and the mutation-specific inhibitor sotorasib in KRAS G12C-mutated cancer (1, 2). Mutational inactivation or genomic deletion of tumor suppressors similarly create vulnerabilities but typically require indirect targeting approaches as exemplified by the synthetic lethal effect of PARP inhibitors in BRCA1/2-mutated cancers (3). A complementary approach relies on exploiting genes that are commonly codeleted with tumor suppressor genes through a synthetic lethality mechanism. The methylthioadenosine phosphorylase (*MTAP*) gene is frequently lost in cancers through codeletion with the *CDKN2A* tumor suppressor gene (4). MTAP is a key metabolic enzyme in the methionine salvage pathway that metabolizes methylthioadenosine (MTA) to adenine and methionine. Using shRNA-based screening platforms, several groups recently defined the Protein Arginine Methyltransferase-5, methylosome protein 50 (PRMT5:MEP50) arginine methyltransferase complex as a selective vulnerability in *MTAP*-deleted cells (5–7). In cells, MTA accumulates upon *MTAP* deletion and was shown to inhibit PRMT5 enzymatic activity, creating a relative PRMT5 activity deficit in *MTAP*-deleted cells.

In eukaryotes, cellular methylation of the terminal guanidino group of arginine in proteins is mediated by three classes of enzymes: Type I enzymes mediate asymmetric dimethylation, symmetric dimethylation is mediated by type II enzymes, whereas type III enzymes monomethylate the guanidino group (8). Arginine methylation broadly regulates a multitude of cellular processes. PRMT5 is the major cellular type II enzyme and regulates gene expression through methylation of multiple substrates, including histones and RNA splicing factors. PRMT5 preferentially methylates arginine within the short consensus sequence “GRG” but derives additional substrate specificity through binding to substrate adaptors, including pICIn, RIOK1, and COPR5 (9, 10). PRMT5 is a MYC transcriptional target and is required to sustain MYC-driven lymphoma maintenance through regulation of RNA splicing (11). Furthermore, PRMT5 is highly expressed in many cancers and broadly promotes oncogenesis, thus providing a rationale for clinical evaluation of PRMT5 inhibition. Several PRMT5 small molecule inhibitors, covering both substrate- and cosubstrate S-Adenosyl methionine (SAM)-competitive mechanisms, have thus been advanced into clinical trials. While these investigations are still ongoing, promising activity has been observed in several cancers, but this activity is offset by an adverse effect profile consistent with an antiproliferative mechanism, including gastrointestinal symptoms and pancytopenia (12, 13). Genetic studies also point to an important role of PRMT5 in normal cellular and organismal biology. PRMT5 KO in mice results in embryonic lethality, and conditional deletion of PRMT5 in the hematopoietic compartment in adult mice demonstrate that PRMT5 is essential for the maintenance of hematopoietic cells (14, 15). Similarly, large-scale CRISPR screens in human cell lines classify PRMT5 as a pan-essential gene (16). Taken together, these data may indicate a narrow therapeutic window of PRMT5 inhibition in oncology.

DNA-encoded chemical library (DEL) technology is a proven and effective screening platform for the discovery of novel small-molecule ligands to disease targets (17). DELs constitute mixtures of small chemical molecules, where each molecule is coupled to a distinctive DNA barcode, capable of informing on the identity of the small molecule. This technology allows screening of compound libraries at an unprecedented scale, with libraries containing up to billions of diverse molecules. One potential limitation of a DEL screen is that hit compounds are isolated by virtue of binding to the target without any assessment of a functional effect on the target during screening. Here, we demonstrate that DEL screens can be directed to identify compounds with a desired mechanism. We reasoned that compounds that preferentially inhibit PRMT5 in the presence of MTA might constitute an attractive modality to treat *MTAP*-deleted cancers with less adverse effects on normal tissues. DEL screening of PRMT5 in the presence of MTA or a SAM analog, sinefungin (SIN) allowed the identification of compounds that depend on a specific cofactor for PRMT5 binding. MTA-dependent compounds inhibit PRMT5 activity in an MTA-cooperative manner and selectively kill *MTAP*-deleted cancer cells while sparing *MTAP* wild-type (WT) cells. While this work was in progress, others also reported the identification of MTA-cooperative PRMT5 inhibitors (18–21).

## Results

**Fig. 1.**
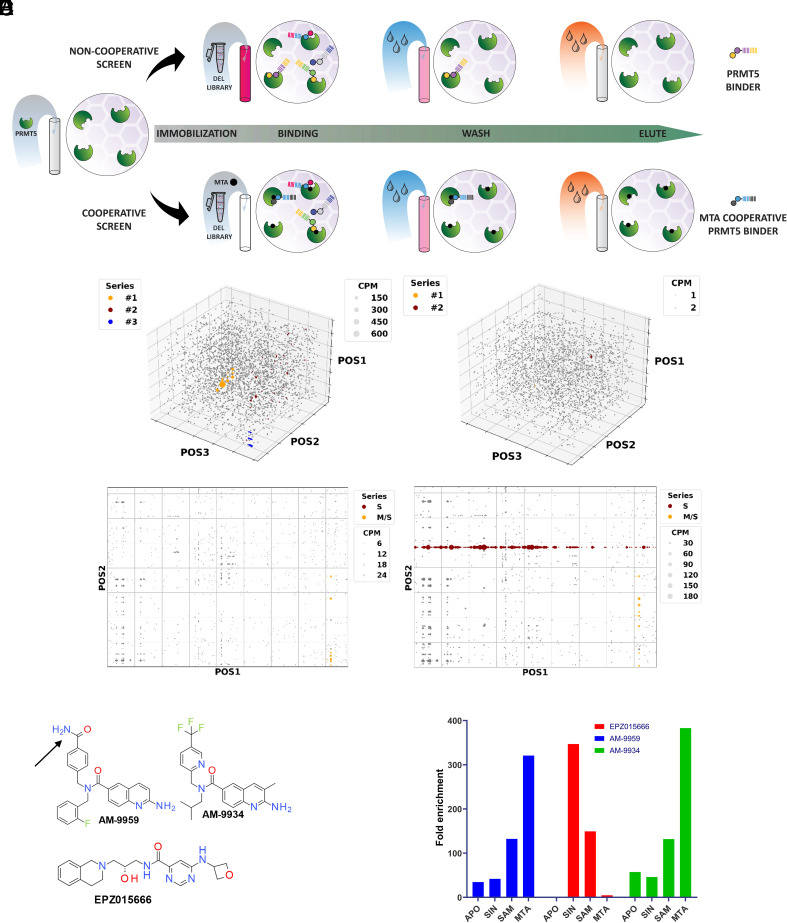
Identification and validation of an MTA-selective PRMT5 inhibitor. (*A*) Screening strategy. (*B* and *C*) Box scatter plots illustrating enriched compounds as a function of their individual building blocks (axes) and relative enrichment (CPM, sequence counts per million reads, dot size). Screens shown are DEL91 (*B*) and DEL123 (*C*) in the presence of MTA (*Left* panels) or SIN (*Right* panels). (*D*) Representative hit compound AM-9959 and structure of EPZ015666 and AM-9934. The arrow denotes point of DNA attachment in the oligonucleotide-conjugated AM-9959 derivative. (*E*) Fold enrichment of oligonucleotide-conjugated compounds from single-round selections in the presence of cofactors as indicated.

We employed DNA-encoded library (DEL) technology to screen for MTA-cooperative inhibitors of PRMT5. HIS-tagged heterotetrameric PRMT5:MEP50 complex (Fig. 1*A*) was incubated with two distinct DELs (*SI Appendix*, Fig. S1), containing a total of 226 million diverse small molecule compounds, in the presence and absence of MTA or SIN, a nonhydrolyzable analog of SAM. SIN was chosen as a substitute for SAM as SAM is subject to spontaneous hydrolysis to MTA, which could potentially confound the screening strategy and output analysis (22). After incubation, nonbinding DEL molecules were removed by washing, and enriched DEL molecules were eluted and subjected to a second cycle of binding-wash-elution, whereafter the identity of enriched molecules was determined using sequencing and barcode deconvolution. After removal of all individual DEL molecules that were observed with less than three independent sequence counts, the remaining DEL molecules were clustered and visualized using scatter plots (Fig. 1 *B* and *C*). The screens from DEL91 resulted in three main chemical series (Fig. 1*B*). DEL91 series 1 (yellow dots) was primarily enriched in the MTA-directed selection, but also showed detectable enrichment in the SIN-directed selection, indicating minimal selectivity of this series. However, this series was later shown to be metabolically unstable despite extensive medicinal chemistry optimization and will not be described further here. DEL91 series 2 (brown dots) did not show any selectivity for the MTA-bound enzyme and will not be described further. In contrast, DEL91 series 3 (blue dots) was only enriched in the MTA selection, indicating selective binding to the MTA-bound form of PRMT5. This series is defined by a single quinoline building block in position 2, and the active binding species in the library was later determined to be a common deletion product from position 3 resulting in the formation of a quinolinic amine (described in detail in ref. 23). The screens from DEL123 resulted in 1 main chemical series, which was enriched exclusively in the SIN selection (Fig. 1*C*, brown dots). This series is defined by the presence of a fragment also present in the known PRMT5 inhibitor EPZ015666/GSK3235025 [hereafter referred to as EPZ015666 (24)]. As this series did not show any enrichment in the presence of MTA, we decided to not pursue this series further and instead use EPZ015666 as an example of compounds from this series. Outside of the EPZ015666 series, DEL123 screening only yielded another minor chemical series (Fig. 1*C*, yellow dots), which showed enrichment both in the presence of MTA and SIN. We synthesized several representatives from this series but were not able to demonstrate any inhibitory activity of the compounds toward PRMT5, and this series will not be described further here.

To validate the selectivity of DEL91 series 3 for the MTA-bound form of PRMT5, we resynthesized a representative hit compound AM-9959 as an oligonucleotide conjugate to mimic a DEL molecule (Fig. 1*D*). As a control, we also synthesized an oligonucleotide-conjugated version of EPZ015666. The two DNA-conjugated compounds were then subjected to a bind-wash-elute cycle using the apo form or cofactor bound forms of the PRMT5:MEP50 complex as baits to mimic the DEL selection conditions, and enrichment of the compounds relative to an unconjugated oligonucleotide was quantified by qPCR using compound-specific primers (Fig. 1*E*). In agreement with previously published data (21), EPZ015666 oligonucleotide conjugate showed no enrichment toward MTA-bound PRMT5 complex and instead showed a strong preference for the SIN-bound complex. In contrast, AM-9959 oligonucleotide conjugate showed a pronounced preference for the MTA-bound complex, with lesser enrichment observed toward the SAM-bound enzyme and very low enrichment in the SIN setting. We speculate that the intermediate enrichment displayed by both compounds toward the SAM-bound complex might reflect the potential contamination of SAM by MTA, as mentioned above.

**Fig. 2.**
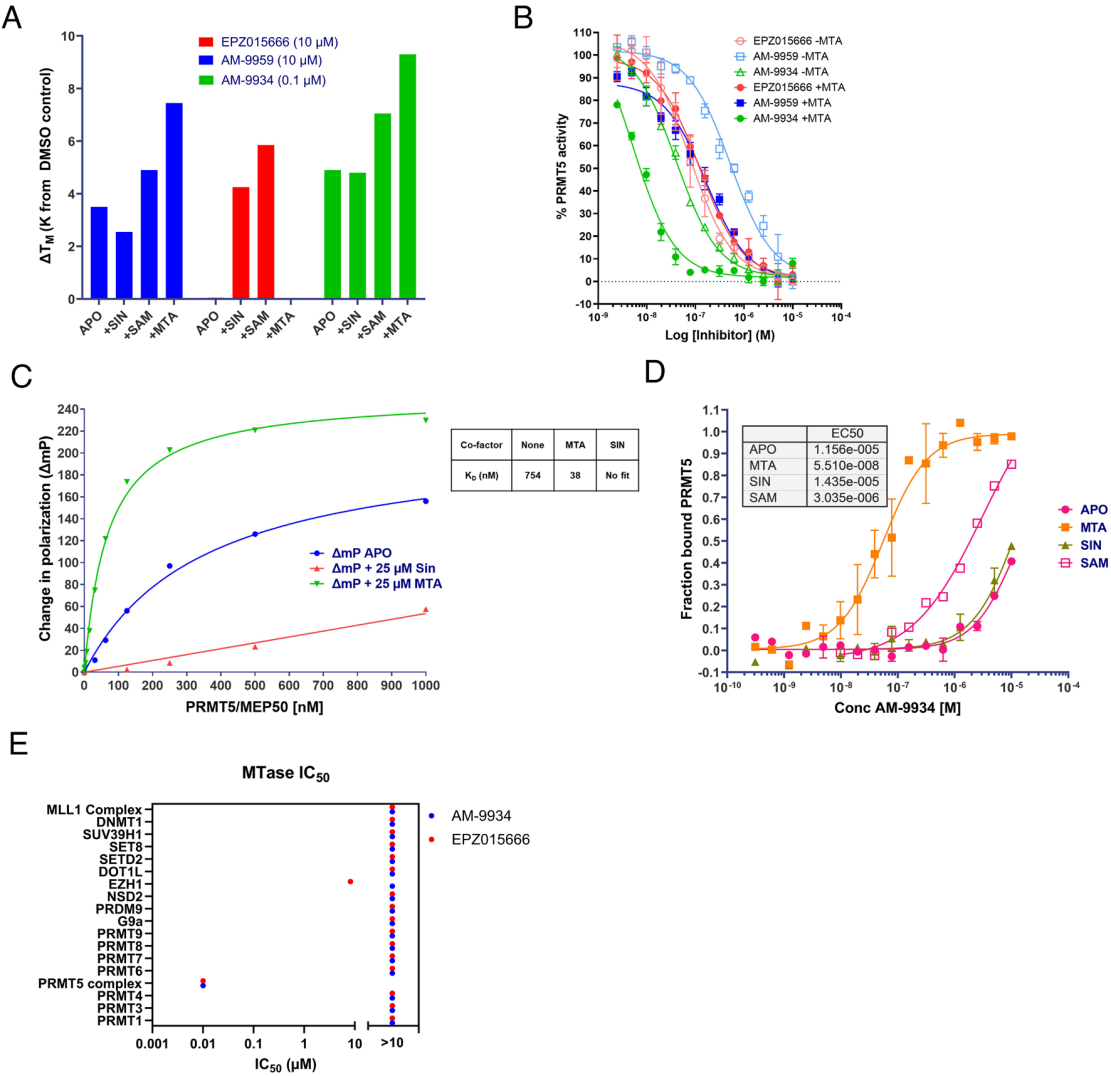
Validation of MTA co-operativity. (*A*) Thermal shift assay showing stabilization of PRMT5:MEP50 complex as a function of compound and cofactor addition. (*B*) Inhibition of PRMT5 methyltransferase activity +/− addition of MTA. (*C*) Fluorescence polarization (FP) assay demonstrating binding of FITC-AM-9934 to PRMT5 after 300 min incubation without or with cofactor in the presence of indicated concentrations of PRMT5:MEP50 complex. *Inset*: K_D_ value of FITC-labeled AM-9934 derivative binding to PRMT5:MEP50 with or without the indicated cofactor as derived from Lineweaver–Burk analysis. (*D*) Microscale Thermophoresis analysis of AM-9934 binding to PRMT5 in the presence of indicated cofactors. (*E*) IC_50_ values of AM-9934 or EPZ015666 against indicated methyltransferase activity. In cases where the IC_50_ was not reached at 10 µM of compound, this is indicated as >10 µM on the secondary X-axis.

We then investigated whether the free compound would retain the same binding characteristics as the DEL hit compound. To this end, we synthesized AM-9959 (Fig. 1*D*) in free form and investigated its binding to PRMT5 using a Thermal Stability Assay (TSA). The PRMT5:MEP50 apo complex without addition of cofactor yielded a single melting point of 54.4 °C. Addition of AM-9959 (10 µM) resulted in a stabilization of the apo complex by 3.5 K, demonstrating direct binding of AM-9959 to the complex (Fig. 2*A*). Addition of either SIN, SAM, or MTA to the PRMT5:MEP50 apo complex resulted in negligible (<0.5 K) stabilization of the complex. Further addition of AM-9959 (10 µM) resulted in the preferential stabilization of the MTA-bound complex (7.5 K), whereas the SIN-bound complex only showed weak stabilization (2.6 K) by AM-9959 and the SAM-bound complex (which also contains some MTA as explained above) showed an intermediary level of stabilization by AM-9959 addition. Taken together, these data indicate that AM-9959 preferentially binds the PRMT5:MEP50 complex in the presence of MTA, although based on these data, we cannot rule out affinity toward other forms of the enzyme. Contrasting with this binding profile, EPZ015666 (10 µM) demonstrated no stabilization of the MTA-bound complex and only showed an interaction with either the SAM- or SIN-bound enzyme (Fig. 2*A*), which is consistent with the oligonucleotide-conjugated compounds.

To investigate whether binding of AM-9959 to the PRMT5:MEP50 complex results in inhibition of PRMT5 enzymatic activity, we measured the catalytic activity of the enzyme in the absence and presence of MTA, and with addition of a range of concentrations of AM-9959 or EPZ015666. As shown in Fig. 2*B*, addition of AM-9959 resulted in inhibition of PRMT5 activity with an IC_50_ of 520 nM. Addition of MTA further potentiated the inhibitory activity of AM-9959 to 160 nM. EPZ015666 also potently inhibited the enzymatic activity of PRMT5 with a measured IC_50_ of 78 nM, but unlike AM-9959, the inhibitory activity of EPZ015666 was not further potentiated by MTA. If anything, inhibition by EPZ015666 was slightly weakened to an IC_50_ of 120 nM. This is consistent with the TSA data presented above.

While AM-9959 possesses the MTA-selective profile that we had set out to identify, it lacks the required potency to be progressed into cellular and in vivo studies. We therefore conducted medicinal chemistry optimization to reach AM-9934 (Fig. 1*D*). When synthesized as an oligonucleotide conjugate, AM-9934 showed a strong preference for the MTA-bound enzyme complex similarly to AM-9959 (Fig. 1*E*). In a TSA experiment (Fig. 2*A*), AM-9934 showed a qualitatively similar binding profile to AM-9959 but with a higher degree of thermal stabilization (9.3 K of stabilization at 0.1 µM of AM-9934 in the presence of MTA, compared to 7.5 K for AM-9959 at 10 µM), indicating an improved affinity of AM-9934 compared to AM-9959. Finally, AM-9934 also showed potent inhibition of PRMT5:MEP50 enzymatic affinity with an IC_50_ of 40 nM which was potentiated to 5 nM in the presence of MTA (Fig. 2*B*). The potency in the presence of MTA may be underestimated by this assay as the measured IC_50_ approximates the concentration of PRMT5:MEP50 complex used in the assay (4 nM of enzyme). Taken together, the data indicate that AM-9934 inhibits PRMT5 in an MTA-cooperative manner with increased potency over AM-9959.

As SAM preparations are contaminated with MTA, SAM-dependent experiments such as PRMT5 catalytic activity are not useful to determine the absolute value of MTA cooperativity of an inhibitor. To estimate the level of MTA cooperativity for AM-9934, we instead sought to measure the affinity of AM-9934 for PRMT5 using orthogonal methods. First, we generated a fluorescently labeled derivative of AM-9934 (*SI Appendix*, Fig. S2), and measured the binding affinity of PRMT5 to this compound using FP. Incubation of 25 nM of FITC-labeled AM-9934 derivative in the absence or presence of MTA or SIN with varying concentrations of PRMT5/MEP50 complex for a range of timepoints (Fig. 2*C* and *SI Appendix*, Fig. S3) was fitted using the Lineweaver–Burke methodology (*SI Appendix*, Fig S3). This analysis yielded a K_D_ of FITC-labeled AM-9934 derivative for the apo enzyme of 754 nM, with a significantly lower K_D_ for the MTA-bound protein of 38 nM. The latter may be an underestimation of the affinity of the AM-9934 derivative for the MTA-bound enzyme as several orthogonal experiments using a lower probe concentration of 1 nM yielded a saturation K_D_ of 9 nM (*SI Appendix*, Fig. S4). Under these conditions, the K_D_ of FITC-labeled AM-9934 derivative was also determined by replotting saturation binding data to fit an association binding experiment and subsequently by a dissociation experiment using free ligand displacement of the FITC-labeled probe. All three methods yielded a comparable K_D_ of approx. 9 nM, which was above the concentration of FITC probe (1 nM) and therefore a more accurate determination of the binding affinity. Incubation in the presence of SIN generated insufficient data for Lineweaver–Burke fitting, indicating low affinity under these conditions. Second, we measured the direct binding of AM-9934 to 25 nM of PRMT5 using microscale thermophoresis (MST) (Fig. 2*D*). In the presence of MTA AM-9934 bound with a K_D_ of 55 nM, whereas binding to either the apo enzyme or the SIN-bound enzyme was much weaker (>10 µM). Taken together, the FP and MST experiments demonstrate that AM-9934 is highly selective for MTA-bound PRMT5. To assess the selectivity of AM-9934 and EPZ015666 for PRMT5 we profiled both compounds against a broad panel of arginine-, lysine-, and DNA-methyltransferases. As shown in Fig. 2*E*, AM-9934 (IC_50_ of 12 nM) and EPZ015666 (IC_50_ of 10 nM) strongly inhibited PRMT5 activity, even without addition of surplus MTA. No significant inhibitory activity was observed toward the other tested methyltransferases demonstrating the high degree of selectivity of AM-9934 toward PRMT5.

**Fig. 3.**
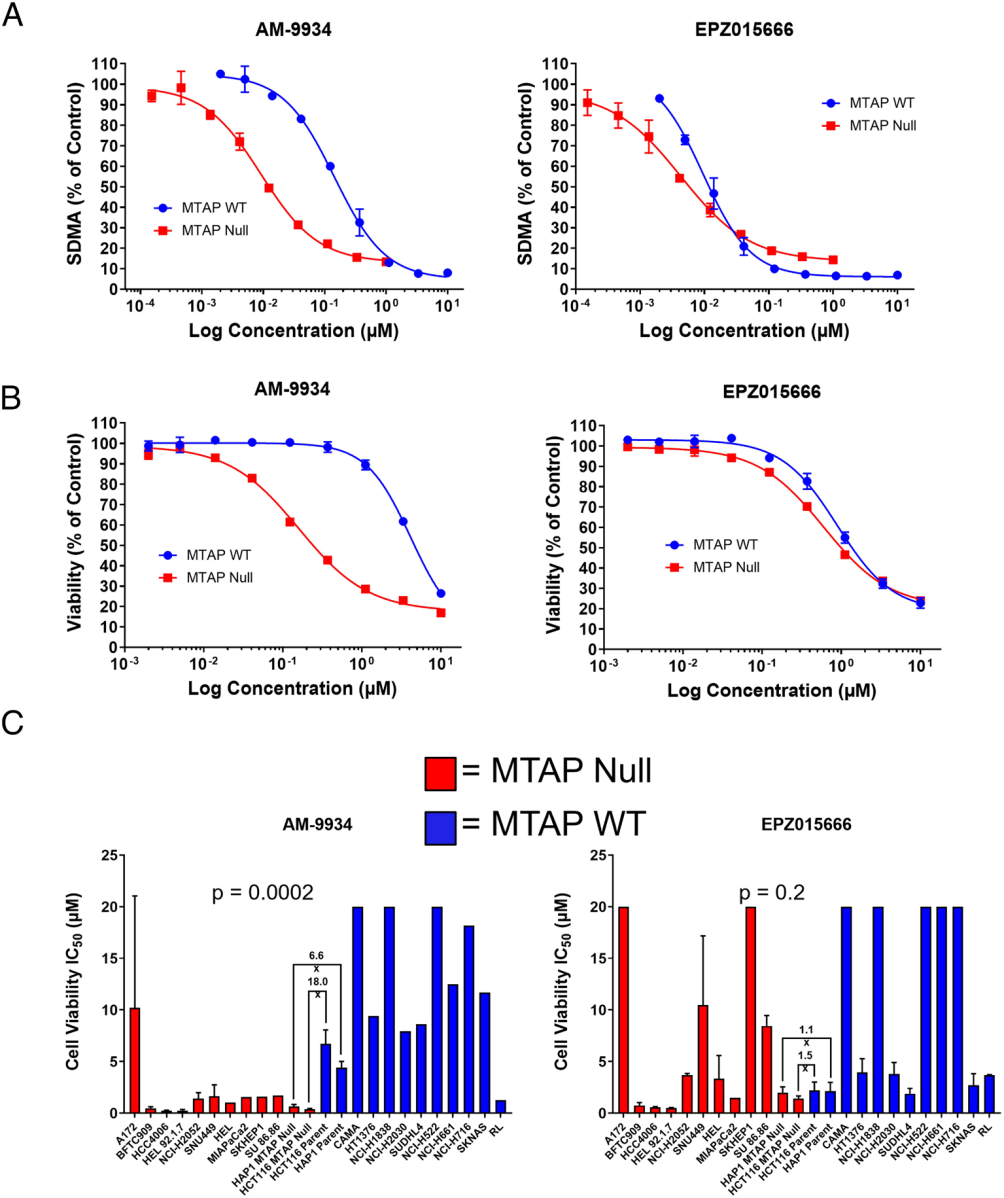
Cellular activity of AM-9934. (*A*) Inhibition of cellular SDMA in HCT116 isogenic cell pair. (*B*) Inhibition of cell viability in HCT116 isogenic cell pair. (*C*) Cell viability IC_50_s across a panel of *MTAP* WT (Blue) and *MTAP^−^*^/^*^−^* (Red) cell lines. *Insets* report fold differences in isogenic cell pairs, *P*-values for WT (Blue) and *MTAP^−^*^/^*^−^* by ANOVA.

To characterize cellular effects of AM-9934, we developed a high-content imaging assay using an antibody against symmetric dimethyl-arginine (SDMA) to assess cellular levels of SDMA after incubation for 3 d with either AM-9934 or EPZ015666. We used an isogenic pair of HCT116 colorectal cancer cell lines that are *MTAP* WT (Parental HCT116 cells) or have been rendered *MTAP*-deleted using CRISPR technology, respectively. This allowed us to assess the impact of *MTAP* status (and hence MTA levels) on AM-9934 and EPZ015666 activity in otherwise identical genetic backgrounds. As shown in Fig. 3*A*, both compounds caused a concentration-dependent reduction of cellular SDMA. However, while EPZ015666 showed little differentiation between parental and *MTAP*-deleted cells (IC_50_ of 9.6 vs. 4.3 nM), AM-9934 showed enhanced potency in the *MTAP*-deleted line (IC_50_ of 9.2 nM) and only weakly reduced SDMA in parental HCT116 cells (IC_50_ of 143 nM) (Fig. 3*A*). Reduction in cell viability following inhibitor treatment followed a similar pattern where EPZ015666 was approximately equipotent between the two cell lines (IC_50_ of 0.60 µM in *MTAP*-deleted cells vs. 0.89 µM in parental cells) whereas AM-9934 was significantly more potent against *MTAP*-deleted cells (IC_50_ of 0.16 µM vs. 4.1 µM in parental cells, Fig. 3*B*). Thrombocytopenia is a known on-target dose-limiting toxicity for the first generation of SAM competitive or SAM cooperative PRMT5 inhibitors that entered clinical development (25). To investigate the effects of AM-9934 on human primary cells, we turned to a human bone marrow–derived megakaryocyte viability assay. AM-9934 treatment inhibited megakaryocyte viability with an IC_50_ of 3.1 µM, which approaches that of parental HCT116 cells, whereas EPZ015666 affected viability in this system with an IC_50_ of 0.36 µM (*SI Appendix*, Figs. S5 and S6). Finally, to investigate the broader potential for AM-9934 to preferentially inhibit viability of *MTAP*-deleted cancer cells, we determined inhibitor effects on viability across a 24-cell line panel harboring both *MTAP* WT and *MTAP*-deleted cell lines from diverse tissue origins. While EPZ015666 did not significantly discriminate between cell lines based on *MTAP* status, AM-9934 showed significantly higher potency toward *MTAP*-deleted cell lines (Fig. 3*C*).

**Fig. 4.**
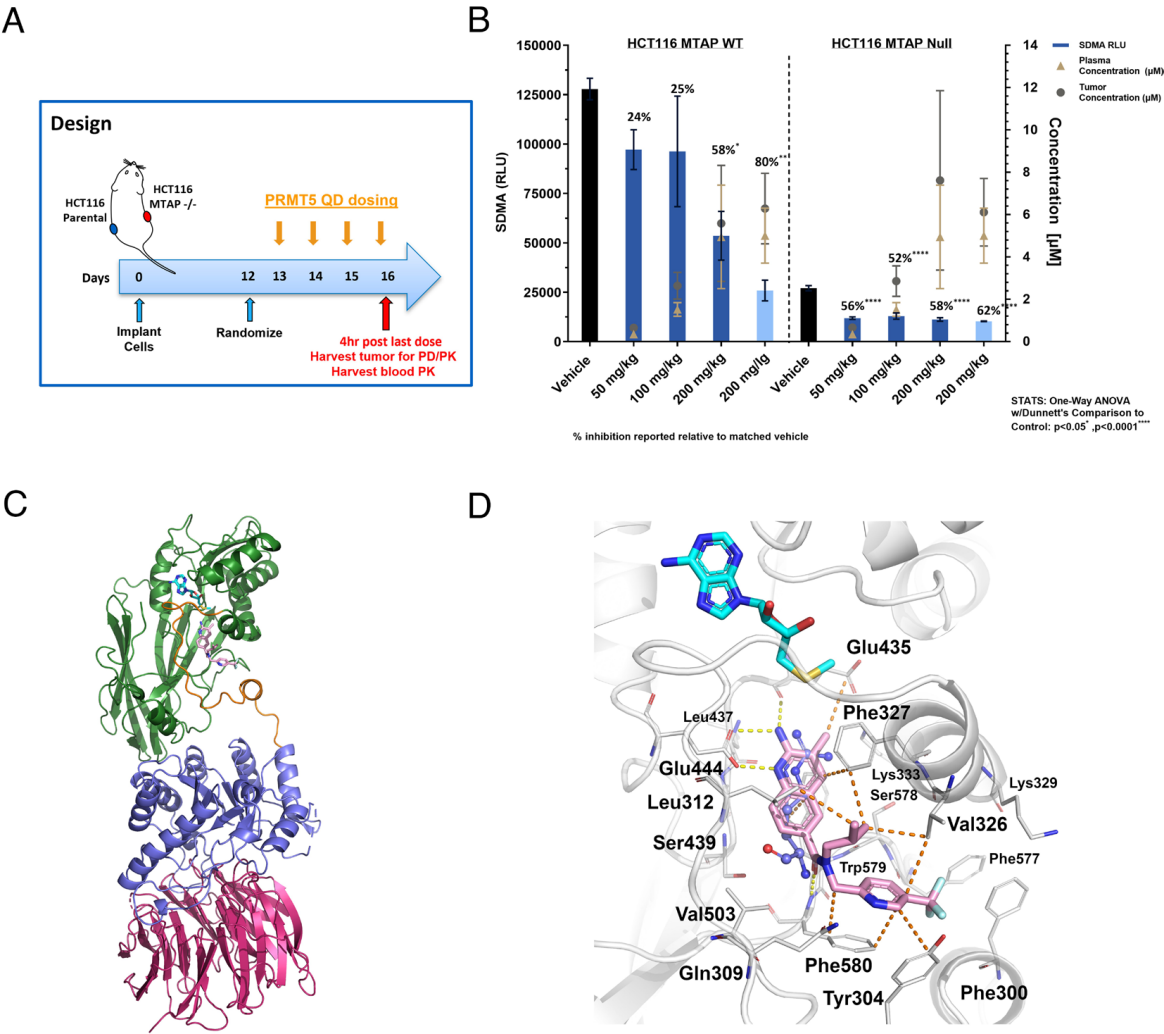
Substantiation of the AM-9934 mechanism of action. (*A*) Design of mouse pharmacodynamic model. Contralateral wt/*MTAP^−^*^/^*^−^* tumors were treated once daily for 4 d as indicated before tumor harvesting for SDMA quantification. (*B*) Tumor SDMA levels (left y-axis) and blood/tumor compound concentrations (right y-axis) 4 h post–last dose of AM-9934 (Dark blue bars) or GSK3326595 (Light blue bars). (*C*) Overall view of AM-9934 in complex with PRMT5 and MTA with secondary structure renditions of MEP50 (warm pink), PRMT5 catalytic domain (green), and the TIM barrel domain (violet), and the loop linking these two domains (orange). Ligands are in stick representation and their carbon atoms are color-coded for AM-9934 (pink) and MTA (cyan). (*D*) Zoom of the substrate/cosubstrate pockets of MTA- and AM-9934-bound PRMT5 with the protein depicted in secondary structure, with key residues depicted in stick representation. The arginine substrate from the superposition of PRMT5:MEP50 in complex with MTA and H4 peptide (PDB: 5FA5) onto the complex has also been rendered (ball and stick). Carbon atoms are color-coded for protein residues (light gray), MTA (cyan), Arginine substrate (violet), and AM-9934 (pink).

Having demonstrated the MTA-selective profile of AM-9934 in vitro and in cells, we then asked whether this profile would translate into selective PRMT5 inhibition in *MTAP*-deleted tumors. Mice were implanted contralaterally on their flanks with parental and *MTAP*-deleted HCT116 cells such that each mouse would carry one WT and one *MTAP*-deleted tumor (Fig. 4*A*). This design eliminates variance in compound exposures between groups and allows direct assessment of *MTAP* status impact on compound effects. Twelve days post–tumor implantation, mice were randomized to treatment groups and treated daily for 4 d with either AM-9934 or GSK3326595, a derivative of EPZ015666 that has progressed into clinical trials. Four hours post the fourth dose, tumors were analyzed for SDMA content by ELISA. As expected, *MTAP*-deleted tumors showed intrinsically lower SDMA levels compared to parental tumors, presumably due to partial PRMT5 inhibition caused by higher MTA levels in *MTAP*-deleted tumors (Fig. 4*B*) (5–7). Treatment with GSK3326595 led to marked suppression of tumor SDMA levels, irrespective of *MTAP* status. Treatment with AM-9934 likewise resulted in suppression of SDMA levels in both tumor types. However, SDMA suppression was insignificant at lower doses in parental tumors and did not approach the suppression levels reached by GSK3326595 treatment even at the highest dose tested, whereas all three doses reached significant levels of SDMA suppression in the *MTAP*-deleted tumors. This confirms the MTA-selective action of AM-9934 in the tumor setting.

To elucidate the binding mode of AM-9934 to PRMT5, we solved the crystallographic structure of this ligand in complex with MEP50:PRMT5 in the presence of MTA. The details of data collection and statistics of refinement can be found in *SI Appendix*, Table S1. In the resulting molecular structure, protein domains are organized as previously observed (26) with the PRMT5-TIM barrel domain in contact with MEP50 on one side and the PRMT5-catalytic domain on the other side (Fig. 4*C*). AM-9934 is located in the catalytic domain in the peptide binding site, where the arginine substrate would bind during the catalytic cycle, and in the vicinity of the coinhibitor MTA (Fig. 4*D*). The 2-aminoquinoline moiety mimics the arginine of the substrate with similar interactions, a salt bridge with Glu444 and a hydrogen bond with Glu435 (6). Moreover, another H-bond is present between the carbonyl of the ligand and the amide of Phe580. The quinoline rings are also in van der Waals contact with residues Glu435, Trp579, and Phe327, and with MTA. In addition, more nonpolar interactions are visible for the rest of AM-9934, where the isopropyl moiety has hydrophobic interactions with Leu312, Val326, and Phe327, while the trifluoro-pyridine group packs against Tyr304, Val326, and Phe580. Residues Tyr304 and Phe580 form a π:π stack with the pyridine ring of the ligand. The molecular structure of the complex allows us to rationalize the preference of AM-9934 for MTA-bound PRMT5. The natural coinhibitor, MTA, is in contact with AM-9934 and its sulfur atom is at 4 Å distance to the ligand primary amine. The presence of an additional methyl group, as in the case of SAM, would result in a steric clash with the ligand, disrupting the formation of the complex.

Taken together, our data demonstrate that selective PRMT5 inhibition in *MTAP*-deleted tumors can be achieved using an MTA-cooperative PRMT5 inhibitor, exemplified by AM-9934.

## Discussion

A current focus in drug development is that of multispecific drugs where the active compound engages not only the target but also one or more additional components to achieve the desired pharmacological outcome (27). One such example is the class of “imid” drugs, exemplified by lenalidomide, which bring cellular proteins, including transcriptions factors IKZF1/3 into proximity of the E3 ubiquitin ligase CRBN, leading to polyubiquitination and subsequent degradation of IKZF1/3 (28). This principle is not limited to small molecule inducers of protein–protein interactions and can be extended across molecular scale to bispecific antibodies, such as the approved medicine blinatumomab (29) which brings CD19-expressing tumor cells into proximity with cytotoxic T cells, leading to tumor cell lysis. Similarly, bispecific molecules might be engineered to induce completely novel interactions or to strengthen existing interactions (30). Here, we used DEL technology to identify molecules that strengthen the low µM interaction between a small molecule metabolite MTA and its target PRMT5. While DEL screening has traditionally been limited to discovery of target binders, we here provide an example of a DEL screening strategy that allowed the direct identification of a hit compound with nM PRMT5 inhibitory activity and the desired feature of MTA co-operativity. Furthermore, modification of the screening setup allowed us to redirect the screen to instead identify analogs of the known SAM-uncompetitive PRMT5 inhibitor EPZ015666. We speculate that the versatile directed binding strategy and large chemical libraries employed by the DEL platform may constitute a particular advantage for discovery of multispecific compounds, as demonstrated by recent proof-of-concept experiments by the Schreiber lab (31).

Medicinal chemistry optimization of AM-9959 to AM-9934 allowed us to profile an MTA-cooperative PRMT5 inhibitor across a diverse set of in vitro and cell-based assays. We initially note several caveats that may confound absolute correlations across the entire portfolio of assays: 1) As noted previously, SAM spontaneously breaks down to MTA (22), and assays that rely on SAM such as the methyl-transferase assay cannot be carried out in a truly MTA-free environment and are therefore not optimally suited for measuring the degree of MTA co-operativity. 2) Appendage of a fluorophore or an oligonucleotide through a linker might alter the affinity of a compound to its target. 3) *MTAP* WT cells contain detectable levels of MTA (5–7). Cooperativity factors may thus not translate completely between cell-free and cellular systems. Cognizant of these limitations, we conclude that AM-9934 binds preferentially to MTA-loaded PRMT5 with high selectivity across orthogonal assay platforms and specifically inhibits PRMT5 in the low nM range in the presence of MTA. We were able to rationalize the selectivity profile of AM-9934 with a molecular structure demonstrating that AM-9934 competes sterically with the substrate arginine and interacts with SAM-competitive MTA. Even in the absence of exogenously added MTA, AM-9934 is strongly selective for PRMT5 versus other arginine- or lysine methyltransferases. In the cellular system, low nM concentrations of AM-9934 decreased SDMA levels after a 3-d incubation only in *MTAP*-deleted cells. This translated to specific inhibition of viability in *MTAP*-deleted cells, although this only occurred at higher concentrations of AM-9934. The existence of an SDMA-specific demethylase has not been firmly established in the literature, and it is conceivable that erasure of the SDMA mark only appears because of protein turnover (9). Others have noted using SAM-competitive PRMT5 inhibition that full suppression of SDMA may require prolonged inhibitor treatment and that effects on viability require near-complete SDMA suppression (32), which agrees with the differential effects observed between PRMT5 suppression by siRNA versus PRMT5 CRISPR knockout (16). We extended these findings in vivo to demonstrate significant inhibition of SDMA in *MTAP^−^*^/^*^−^* tumors across a range of doses, while sparing SDMA in *MTAP* WT tumors except at the highest dose tested. Even at 200 mg/kg dosing, suppression in WT tumors did not approach that in *MTAP^−^*^/^*^−^* tumors in absolute levels. In *MTAP^−^*^/^*^−^* tumors, suppression was similar across the dose range even though exposures increased with dose (Fig. 4*B*), suggesting that a maximum level of attainable suppression had been achieved. The remaining SDMA signal might be explained by limited duration of dosing (3 d 4 h) and the potential presence of *MTAP*-expressing stromal cells in the samples (32, 33).

In summary, using a cofactor-directed DEL screening strategy, we identified a series of MTA-cooperative PRMT5 inhibitors and provide in vitro and in vivo evidence supporting this mechanism as an attractive therapeutic strategy in *MTAP^−^*^/^*^−^* tumors. Other preclinical and early clinical stage MTA-cooperative PRMT5 inhibitors from diverse chemotypes provide further support for this modality (18–21). We speculate that the therapeutic index of MTA-cooperative PRMT5 inhibitors might ultimately be defined by the difference in MTA concentrations between tumor and normal tissues. Agents that modify cellular MTA levels or the ratio between SAM and MTA might offer rational combination opportunities with MTA cooperative PRMT5 inhibitors. One such combination is now being tested in the clinical setting (34). Further optimization of AM-9934 ultimately led to the development of the clinical candidate AMG 193 which has demonstrated initial antitumor responses in a first-in-human clinical study (NCT05094336) in patients with *MTAP* null tumors (35).

## Materials and Methods

### Cell Lines.

Parental (HD PAR-034) and *MTAP^−^*^/^*^−^* (HD R02-033) colorectal cancer HCT116 cell lines were obtained from Horizon Discovery and maintained in RPMI1640 supplemented with 10% fetal bovine serum and antibiotics or in McCoy 5A supplemented with 10% fetal bovine serum (for mouse experiments). Other cell lines were obtained from ATCC and maintained according to instructions. Effects of compounds on cell viability were assessed using the CellTiter-Glo Kit from Promega (G7571) after 6 d of compound incubation and quantified using an EnVision® 2105 multimode plate reader (PerkinElmer) in luminescence mode.

### Reagents and Antibodies.

Human heterotetrameric PRMT5:MEP50 complex consisting of recombinant insect cell baculovirus coexpressed N-term FLAG-tagged PRMT5 and N-term His-tagged MEP50 was obtained from Reaction Biology Corp (HMT-2-148). Anti-HIS purification resin was obtained from R&D systems (IP999). MTA, SIN, and EPZ015666 were obtained from Sigma. Histone H2A was obtained from BPS Biosciences (52021). Antibodies directed against SDMA (SDMA 13222s) and GAPDH (2118) were obtained from Cell Signaling Technology. DELs were synthesized as described previously by a tagged-split-and-pool chemistry approach (36).

### Mice.

All animal experimental protocols were approved by the Amgen Institutional Animal Care and Use Committee (IACUC) and were conducted in accordance with the guidelines set by the Association for Assessment and Accreditation of Laboratory Animal Care. Mice were housed in an environmentally controlled room (temperature 23 ± 2 °C, relative humidity 50 ± 20%) on a 12-h light/dark cycle. Mice were fed commercial rodent chow and water ad libitum. Mice with a tumor size exceeding 2,000 mm^3^ were removed from the study and euthanized (37). Mice were housed in sterilized filter-capped cages and maintained under aseptic and pathogen-free conditions. All studies used female 4- to 8-wk-old athymic nude, (Charles River Laboratories). AM-9934 was formulated in 2% hydroxypropyl methylcellulose and 1% Tween-80 at pH2.0. AM-9934 was stored at 2 to 8 °C and protected from light. AM-9934 was mixed well before PO administration.

### Selections Using DELs and Oligonucleotide-Conjugated Compounds.

DNA-encoded library material and/or small molecule–oligonucleotide conjugates were vacuum dried and afterward resuspended in a solution consisting of 6 µM PRMT5/MEP50 complex supplemented with 60 µM of either MTA or SIN in a buffer consisting of 30 mM Tris-HCl pH 7.5, 150 mM NaCl, 1 mM TCEP, 0.005% Tween-20, 0.5 mg/mL herring-sperm carrier DNA, and 0.1% BSA and incubated for 30 min. Following incubation, PRMT5/MEP50 was immobilized using 20 µL Anti-HIS purification resin, incubated for 5 min, then washed with 5 × 80 µL selection buffer under vigorous agitation, and then drained by vacuum to remove unbound library molecules. Bound molecules were then eluted by addition of 50 µL 72 °C-preheated TBS supplemented with 2 mM TCEP for 15 min, and the eluate was collected by centrifugation. The purified material was either quantified by qPCR (small molecule–oligonucleotide conjugates) or subjected to an additional round of affinity selection with fresh PRMT5:MEP50 complex and then subjected to next-generation DNA sequencing to quantify DEL barcodes, as previously described (36).

### Thermal Shift Assay.

0.1 µM PRMT5:MEP50 complex was incubated with compounds in a buffer consisting of 50 mM Tris-HCl pH 8.0, 50 mM NaCl, 1 mM TCEP, and 4xSypro Orange (Thermo Fisher, S-6650) in the absence or presence of cofactor (10 µM SIN, 10 µM SAM, or 5 µM MTA) and read on a StepOne™ Real-Time PCR machine (Applied Biosystems™) using a temperature ramp of 0.5 °C/min over a range of 25 to 95 °C.

### FP Assay.

FITC-labeled AM-9934 derivative was incubated in a buffer consisting of 50 mM Tris-HCl pH 8.0, 50 mM NaCl, 0.01% Tween-20, and 1 mM TCEP with a range of PRMT5/MEP50 complex concentrations in the absence or presence of cofactor (25 µM of SIN or MTA) and mP values were read immediately and then at various time points up to 3 h. The binding of probe (ΔmP) was plotted as a function of protein concentration in GraphPad Prism 10 and saturation K_D_ was derived using the equation: One site − Specific binding, with the binding model: Y = Bmax*X/(Kd + X). The data were replotted as ΔmP as a function of time in minutes for each protein concentration used, and k_obs_ for each protein concentration was derived using the equation: One phase association using the model: Y = Y0 + (Plateau − Y0) * (1 − exp(−K * x)). The derived k_obs_ values (min^−1^) were plotted as a function of protein concentration and the following parameters were determined: dissociation constant k_d_ (intersection with the y-axis, min^−1^) and association constant k_a_ (slope). From these constants, the K_D_ was determined. For dissociation experiments, 1 nM of FITC-labeled AM-9934 derivative and 20 nM of PRMT5/MEP50 complex were allowed to equilibrate for 2 h at room temperature before dissociation was initiated by the addition of 30 µM of AM-9934 and mP values were read immediately and at various time points for up to 22 h. The dissociation rate k_d_ was calculated using the equation: Y = (Y0 − Plateau) * exp(−K * X) + Plateau. The association rate was calculated using the equation: k_a_ = (k_d_)/K_D_ = 1.0 * 10^4^ (M^−1^s^−1^) and the K_D_ was derived as k_a_/k_d_.

### PRMT5 Methyltransferase Assays.

Inhibition of PRMT5:MEP50 protein methyltransferase activity was measured using the MTase-Glo™ Methyltransferase Assay kit from Promega, according to the manufacturer’s protocol. In short, a reaction volume of 8 µL consisting of 4 nM PRMT5:MEP50, 5 µM SAM, 2.5 µM FL Histone H2A in the absence or presence of 1 µM MTA was incubated overnight, and protein activity/inhibition signal was developed by addition of MTase Glo™ Reagent and MTase-Glo™ Detection Solution according to the manufacturer’s protocol. End-point luminescence was read on a Perkin Elmer EnVision® 2105 multimode plate reader. Profiling of AM-9934 and EPZ015666 across a panel of human arginine-methyltransferases was performed by BPS Bioscience profiling services.

### Microscale Thermophoresis.

Monomeric His-tagged PRMT5 (Sino Biological, 1074-H18H) was labeled at a concentration of 50 nM with 25 nM RED-Tris NTA A650 dye from Nanotemper (L018). Labeled PRMT5 was then incubated with compound in the absence or presence of cofactor (final concentration of 10 µM of MTA or SIN) in Premium coated capillaries (Nanotemper) and analyzed on a Monolith Dianthus (Nanotemper) reader using the Nano Red sensor at medium laser power.

### SDMA Imaging Assay.

Cells were seeded into 96-well plates and incubated with compound for 3 d before fixing with 4% PFA (in 1× PBS) for 15 min. Cells were then washed once with Wash Buffer (1× PBS + 1% BSA + 0.2% Triton X-100), 100 µL/well for 15 min before blocking for 1 h Using Licor Odyssey blocking reagent (927 to 40,000) (100 µL/well). After three washes in Wash Buffer, cells were then stained for 2 h with anti-SDMA antibody (1:2,000 in Wash Buffer), washed three times in Wash Buffer, stained for 1 h in the dark using anti-rabbit Alexa-488 (1:2,000 in Wash Buffer) and Hoechst 33342 (1:5,000), followed by three washes in Wash Buffer before quantifying on a Cellomics Array Scan image system.

### In Vivo Contralateral Tumor Model.

HCT116 colon carcinoma WT and *MTAP^−^*^/^*^−^* isogenic cells were determined to be free of contamination with mycoplasma as well as a panel of murine viral pathogens in addition to being authenticated. Mice were inoculated s.c. with 2 × 10^6^ HCT116 WT cells on the left flank and 2 × 10^6^ HCT116 *MTAP^−^*^/^*^−^* cells on the right flank in 33% Matrigel. Mice were enrolled once both tumors reached 250 to 500 mm^3^ and randomized into vehicle vs. PRMT5i treated groups (N = 3). Vehicle or PRMT5i treatment started on day 14 postimplantation orally and treated once daily for 4 d. Blood was collected at 4 h post–last dose (day 17 postimplantation) for pharmacokinetic analysis in Microtainer tubes containing lithium heparin. Blood was centrifuged at 7,000 rpm at 4 °C for 7 min. Tumors from both sides were also collected and snap-frozen for pharmacokinetic analysis and SDMA ELISA. Plasma and tumors were collected and stored at −80 °C until analysis.

### Tumor Protein Lysates.

Preparation of protein lysates from snap-frozen tumors was carried out as follows: samples were collected in a Covaris Tissue Tube (520001 TT1) to be used in conjunction with the CryoPrep. Tumor samples were snap-frozen in liquid nitrogen and placed on dry ice. Prior to pulverization, a transfer tube (520010 TC13 13 × 65 mm tube [glass]) was attached into the top TT1 tube assembly and then placed into the CryoPrep where the sample was pulverized using a hammer and anvil mechanism. The pulverized sample was then transferred into the glass tube and placed on dry ice. The sample was then lysed using RIPA buffer (50 mM Tris-HCl, pH 7.5, 1% Igepal, 0.5% Sodium Deoxycholate, 150 mM NaCl, 0.1% SDS) supplemented with 1x HALT Phosphatase & Phosphatase Inhibitor. Then, freshly diluted 1x lysis buffer was added at 1 mL/sample tissue, and previously pulverized tumor tissue was resuspended. Samples were lysed on ice for 30 min. The suspension was transferred to a 2 mL Eppendorf tube and then centrifuged at 14,000 RPM at 4 °C for 10 min. The supernatant was then transferred into a new 2 mL Eppendorf tube. The protein concentration of each lysate was then determined using a BCA assay. Lysates were normalized for total protein/well and loaded onto MSD plates at 480 ng/well in lysis buffer for SDMA ELISA plate.

### SDMA ELISA.

100 µL of lysates (50 ng/well in duplicates) was transferred to the 96-well high binding plates, and plates were then shaken for 2 h at RT (room temperature) and then washed four times with 200 µL 1× PBS-T. SuperBlock Solution (Thermo Scientific) was added 150 µL per well into the SDMA plates. Plates were then shaken for 2 h at RT and then washed four times with 200 µL 1× PBS-T. Then, 100 µL of the anti-sDMA antibody diluted 1:2,000 in 1×PBS-T + 1% BSA was added to the wells and incubated while covered overnight at 4 °C. Plates were washed four times with 200 µL 1×PBS-T, and then, 100 µL per well of secondary antibody (anti-rabbit IgG horseradish peroxidase conjugate, 1:4,000 diluted in 1×PBS-T + 1% BSA) was added and incubated while covered for 1 h at RT while shaking. Plates were washed four times with 200 µL 1xPBS-T, and then, 100 µL per well of lumunata Read Buffer was added and incubated while covered for 15 min before each plate was analyzed immediately on Envision reader (PerkinElmer) using luminescence detection. The lysates were normalized for total protein, and signals were then used to show the treatment group levels SDMA protein inhibition level compared to the vehicle group in 50 ng of a given tumor sample.

### X-Ray Crystallography.

PRMT5/MEP50 protein complex for X-ray crystallography was recombinantly expressed in a baculovirus expression system according to literature procedures (26). PRMT5/MEP50 protein complex at 13.5 mg/mL was mixed 1:1.2:1.2 molar excess ratio of MTA and AM-9934 in 50 mM Tris pH 7.5, 500 mM NaCl, 10% Glycerol, 1 mM TCEP and incubated on ice for 15 min prior to crystallization set-up. Protein crystallization was performed using the sitting drop vapor diffusion technique in 96-well trays at 8 °C using a Mosquito. Crystals of the PRMT5–MEP50–MTA–AM-9934 complex grew from a 1:1 mix of this protein complex in 1 M Bis-Tris pH 5.5, 10 to 25% PEG3350, 100 to 250 mM MgCl_2_ over a period of 1 wk. These crystals were harvested in 0.1 M Bis-Tris pH6, 15% PEG3350, and 200 mM MgCl_2_ using 25% glycerol as cryoprotectant, and flash-frozen in liquid N_2_ at 100 K for data collection. All datasets were collected on a Pilatus3 6 M silicon pixel detector at the Advanced Light Source Beamline 5.0.2 at wavelength 1.00000 Å and temperature 100 K. The data were integrated and scaled using HKL2000 (38). The structures were solved by molecular replacement using Phaser (39) from the CCP4 program suite (40) with 6CKC as a search model. The structures were refined using Phenix (41). The structure of PRMT5-MEP50 in complex with MTA and AM-9934 was determined at 3.1Å resolution with R-factor R_work_ of 21.6% and R_free_ of 27.2% (PDB ID: 9NWY).

## Supplementary Material

Appendix 01 (PDF)

## Data Availability

The structure of the PRMT5–MEP50–MTA–AM-9934 complex (PDB ID: 9NWY) (42) has been deposited in the PDB database, and the authors will release the atomic coordinates upon publication.
