# Supplementary material for: MTA-cooperative PRMT5 inhibitors from cofactor-directed DNA-encoded library screens
Source: Proc Natl Acad Sci U S A. 2025 May 16;122(20):e2425052122. doi: 10.1073/pnas.2425052122 (PMC12107103; doi:10.1073/pnas.2425052122)
Supplement: Supplementary file 1 — Appendix 01 (PDF) [file pnas.2425052122.sapp.pdf]

Supporting information for Andersson et al.

Chemistry:

Layout of DELs used.

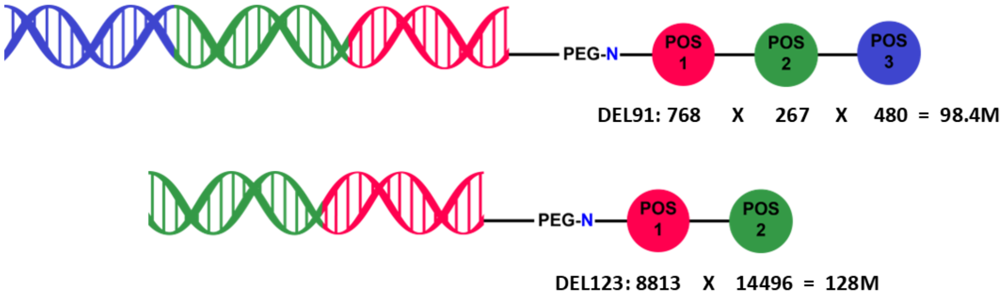

Figure S1. Composition and architecture of DELs screened. POS1-3 denotes the different positions used during library synthesis, matching colors denote corresponding barcodes, and numbers below the individual positions correspond to the number of diverse building blocks used for each position.

General Reductive Amination protocol.

A solution of the relevant aldehyde (1.0-1.2 equiv) and the relevant 1°-amine (1.0-1.2 equiv) in MeOH was stirred for 2 hours at RT. The solution was then cooled to 0°C and $NaCNBH_3$ (1.3 equiv) was carefully added in portions and the reaction was thereafter stirred at RT for 16 hours. Ice water was added to quench the reaction and the mixture was concentrated under reduced pressure. The crude 2°-amine was then purified as indicated.

General Amide Formation protocol.

HATU (1.25 equiv) was added to a mixture of 2-amino-3-methylquinoline-6-carboxylic acid (1.0-1.1 equiv), the relevant 2°-amine (1.0-1.1 equiv) and TEA or DIPEA (3-5 equiv) in dry DMF at RT. The reaction was stirred at RT at 45 °C for 2-16 hours, whereafter H_2O and EtOAc were added. The layers were separated, and the organic layer was washed with NaOH (1 M) or aq sat $NaHCO_3$, dried over Na_2SO_4, filtered, and concentrated under reduced pressure. The residue was purified, as indicated, to yield the final compound.

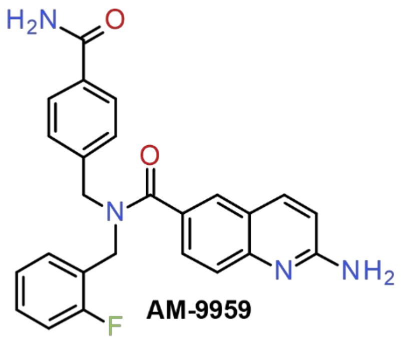

AM-9959

Synthesis of *2-Amino-N-(4-carbamoylbenzyl)-N-(2-fluorobenzyl)quinoline-6-carboxamide* (AM-9959).

4-(((2-Fluorobenzyl)amino)methyl)benzamide (**S1**).

Reductive amination between 4-formylbenzamide and (2-fluorophenyl)methanamine was purified by Flash CC (C18, eluting with MeCN 5-95% in H_2O, with 0.1% formic acid as an additive) to **S1** (120 mg, 0.46 mmol, 14 %). *m/z* (ESI): 259.1 $(M+H)^+$. Amide formation between **S1** and 2-aminoquinoline-6-carboxylic acid was purified by Flash CC (Amino D, eluting with 0-100% EtOAc in heptane, followed by 0-20% MeOH in EtOAc) to yield **AM-9959** (38 mg, 89 µmol, 46% yield).^{1}H NMR (DMSO-d_6, 400 MHz, 330 K) δ 7.9-7.9 (m, 1H), 7.84 (td, 2H, *J*=2.0, 8.2 Hz), 7.77 (d, 1H, *J*=1.9 Hz), 7.5-7.6 (m, 1H), 7.45 (td, 1H, *J*=0.6, 8.6 Hz), 7.3-7.4 (m, 4H), 7.1-7.2 (m, 2H), 6.80 (d, 1H, *J*=8.9 Hz), 6.48 (s, 2H), 4.65 (s, 2H), 4.63 (s, 2H). ESI-HRMS: calcd for $C_{25}H_{22}FN_4O_2$: m/z. $[M + H]^+$ = 429.1721; found $[M + H]^+$ = 429.1722.

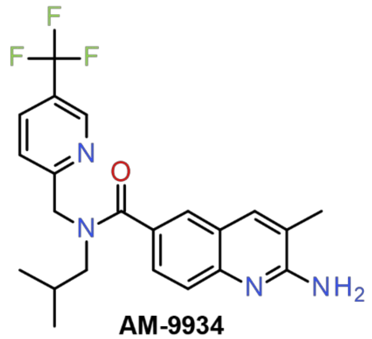

AM-9934

Synthesis of *2-Amino-N-isobutyl-3-methyl-N-((5-(trifluoromethyl)pyridin-2-yl)methyl)quinoline-6-carboxamide* (AM-9934).

2-amino-3-methylquinoline-6-carboxylic acid (**S3**).

Under a N_2 atmosphere, a solution of KtBuO (1.0 M in THF, 0.84 L, 0.84 mol) was added to a stirred solution of propionitrile (60 mL, 0.84 mol) in DMSO (0.75 L) at 0°C. After stirring the mixture for 15 min, methyl 4-amino-3-formylbenzoate (75 g, 0.42 mol) was added in portions, and then heated at 50°C for 16 hours. The reaction mixture was cooled, diluted with H_2O (1.0 L), extracted with EtOAc (2 x 1.0 L). The aqueous layer was adjusted to pH 6.5 with HCl (1.5 M). The resulting solid was filtered,

washed with H_2O (2 x 1.0 L), acetone (2 x 1.0 L), and dried under vacuum overnight. The solid was stirred in MTBE (1.0 L) for 12 hours, filtered, and concentrated under reduced pressure to provide **S3** (52.3 g, 260 mmol, 62% yield). ^{1}H NMR (DMSO-d_6, 400 MHz) δ 10.0 (br s, 1 H), 8.24 (d, 1H, *J* = 2.0 Hz), 7.91 (dd, 1H, *J* = 8.7, 2.0 Hz), 7.86 (s, 1 H), 7.46 (d, 1H, *J* = 8.7 Hz), 6.67 (s, 2 H), 2.22 (s, 3 H). *m/z* (ESI): 203.0 $(M+H)^+$.

2-Methyl-N-((5-(trifluoromethyl)pyridin-2-yl)methyl)propan-1-amine (**S4**).

Reductive amination between 5-(trifluoromethyl)picolinaldehyde (3.0 g, 17.3 mmol) and isobutylamine (1.5 g, 20.2 mmol) was purified by Flash CC (SiO_2, eluting with EtOAc/EtOH (3/1) 0-100% in heptane) to afford **S4** (2.81 g, 12.1 mmol, 70% yield) as a brown oil. ^{1}H NMR ($CDCl_3$, 400 MHz) δ 8.81 (s, 1H), 7.88 (dd, 1H, *J*=8.1, 2.1 Hz), 7.50 (d, 1H, *J*=8.1 Hz), 3.98 (s, 2H), 2.46 (d, 2H, *J*=6.6 Hz), 1.73 - 1.84 (m, 2H), 0.94 (d, 6H, *J*=6.6 Hz). *m/z* (ESI): 233.0 $(M+H)^+$.

Amide formation between **S3** and **S4** was purified by Flash CC (SiO_2, eluting with 20-90% EtOAc in heptane) to give **AM-9934** (177 mg, 0.43 mmol, 82% yield) as a light-yellow solid. ^{1}H NMR (DMSO-d_6, 400 MHz, 340 K) δ 8.9-8.9 (m, 1H), 8.14 (dd, 1H, *J*=1.8, 8.2 Hz), 7.74 (s, 1H), 7.65 (d, 1H, *J*=1.3 Hz), 7.5-7.6 (m, 1H), 7.46 (d, 1H, *J*=8.6 Hz), 7.43 (dd, 1H, *J*=2.0, 8.5 Hz), 6.23 (s, 2H), 4.81 (s, 2H), 3.28 (d, 2H, *J*=7.4 Hz), 2.2-2.2 (m, 3H), 1.9-2.0 (m, 1H), 0.82 (br s, 6H). ESI-HRMS: calcd for $C_{22}H_{24}F_3N_4O$: m/z: $[M + H]^+$ = 417.1897; found $[M + H]^+$ = 417.1896.

Synthesis of a FITC-labelled derivative of AM-9934

To synthesize a FITC-labelled derivative of AM-9934, we proceeded through initial synthesis of Methyl 4-[(isobutylamino)methyl]benzoate:

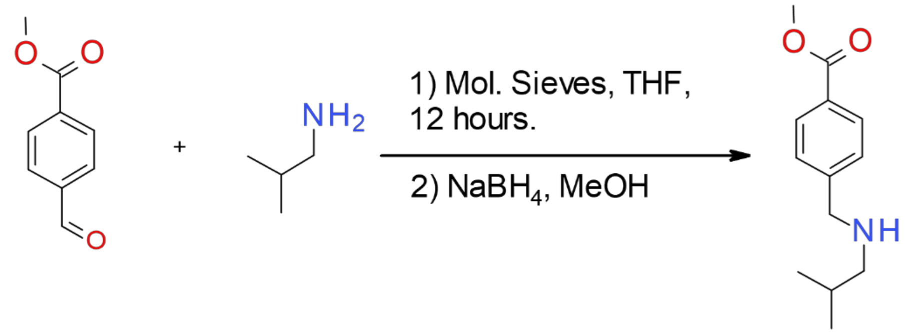

To a solution of 4-formyl-benzoic acid methyl ester (2.3 gram, 14 mmol) and 2-methylpropan-1-amine (1.025 g, 14 mmol) in dry THF (10 mL) was added approx. 30 oven-dried molecular sieves (3 Å) and reaction was shaken at 22°C for 16 hours. To the reaction mixture was added 2 mL dry MeOH followed

by sodium borohydride (0.530 g, 14 mmol) in small portions over 5 minutes. Reaction mixture was stirred for 60 minutes and poured into 50 mL 10% $KHSO_4$ in water and stirred for 10 min. Combined solvent added 1 M $NaHCO_3$ (aq.) until basic, and extracted with 2x100 mL EtOAc. Organic phase was washed with brine (20 mL), dried over (Na_2SO_4, 3 gram), filtered, and evaporated under reduced pressure to yield crude methyl 4-[(isobutylamino)methyl]benzoate compound (550 mg, 18 %), which was used directly in next step.

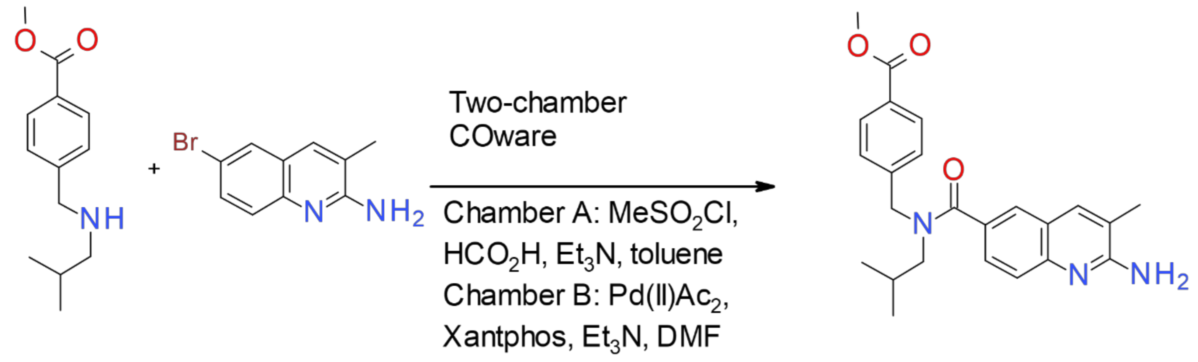

Methyl 4-[[(2-amino-3-methyl-quinoline-6-carbonyl)-isobutyl-amino]methyl]benzoate:

To chamber A of a 2x20 ml two chamber COware gas reactor was added formic acid (151 µL, 4.0 mmol) and methanesulfonyl chloride (309 µL, 4.0 mmol) in 4 mL toluene and to chamber B was added methyl 4-[(isobutylamino)methyl]benzoate (531 mg, 2.4 mmol), 6-bromo-3-methyl-quinolin-2-amine (474 mg, 2.0 mmol), Xantphos (57.6 mg, 0.1 mmol) and triethylamine (405 mg, 4.0 mmol) in 4 mL dry DMF. Reaction chambers were degassed by three evacuation and Argon filling cycles and palladium(II)acetate was added to chamber B and followed by triethylamine (1.12 mL, 8.0 mmol) to chamber A. Reaction was heated to 90°C for 3 hours. The cooled chamber B was poured into 150 mL EtOAc, and washed with 2x15 mL LiCl (5%, aq.), brine (15 mL), dried over (Na_2SO_4, 3 gram), filtered and evaporated under reduced pressure to yield crude product, which was purified by flash chromatography to yield 230 mg (28%) of methyl 4-[[(2-amino-3-methyl-quinoline-6-carbonyl)-isobutyl-amino]methyl]benzoate with an UV purity >98 % at 254 nm.

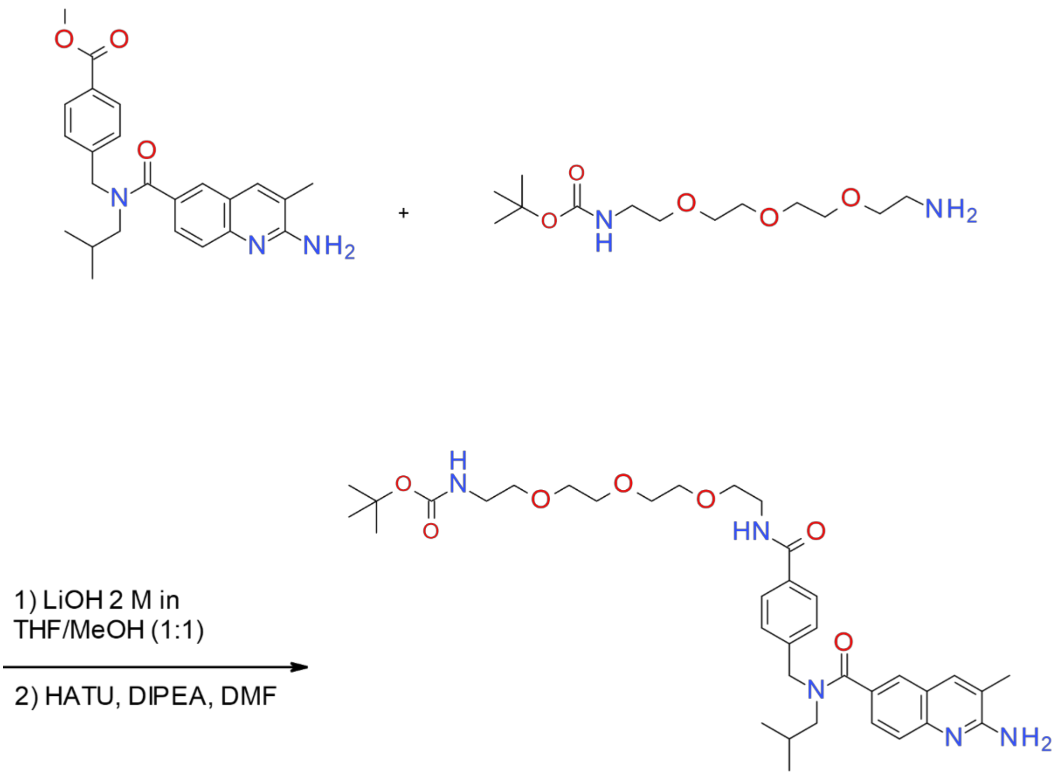

Tert-butyl N-[2-[2-[2-[2-[[4-[[(2-amino-3-methyl-quinoline-6-carbonyl)-isobutyl-amino]methyl]benzoyl]amino]ethoxy]ethoxy]ethoxy]ethyl]carbamate:

To a solution of methyl 4-[[(2-amino-3-methyl-quinoline-6-carbonyl)-isobutyl-amino]methyl]benzoate (230 mg, 0.56 mmol) in a 1:1 mixture of THF and methanol (6 mL) was added 560 µL of a 2 M LiOH (aq. 1.13 mmol). Reaction mixture was stirred at 50°C for 2 hours and to the cooled solution was added 280 µL 2 M HCl (aq.) and reaction mixture evaporated under reduced pressure. The crude was dissolved in 5 mL dry DMF and added tert-butyl N-[2-[2-[2-(2-aminoethoxy)ethoxy]ethoxy]ethyl]carbamate (199 mg, 0.68 mmol), HATU (237.2 mg, 0.624 mmol) and DIPEA (296 µL, 1.7 mmol) and stirred for 2 hours before being poured into 150 mL EtOAc and washed with 2x15 ml LiCl (5 %, aq.), brine ((25 mL), dried over(Na_2SO_4, 3 grams), filtered and evaporated under reduced pressure to yield crude product which purified by flash chromatography to yield 55.5 mg (15 %) of tert-butyl N-[2-[2-[2-[2-[[4-[[(2-amino-3-methyl-quinoline-6-carbonyl)-isobutyl-amino]methyl]benzoyl]amino]ethoxy]ethoxy]ethoxy]ethyl]carbamate with a UV purity >95 at 254 nm.

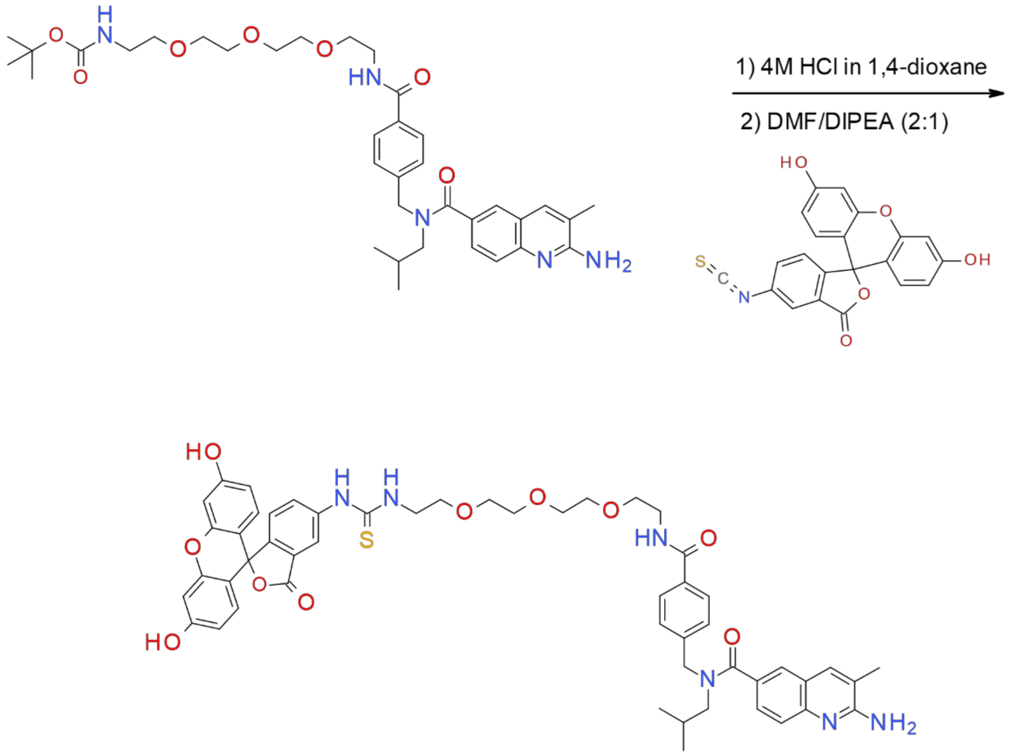

5-[2-[2-[2-[2-[[4-[[(2-amino-3-methyl-quinoline-6-carbonyl)-isobutyl-amino]methyl]benzoyl]amino]ethoxy]ethoxy]ethoxy]ethylcarbamothioylamino]-2-(3-hydroxy-6-oxo-xanthen-9-yl)benzoic acid:

To 4 mL of a 4 M HCl in 1,4-dioaxene solution was added tert-butyl N-[2-[2-[2-[2-[[4-[[(2-amino-3-methyl-quinoline-6-carbonyl)-isobutyl-amino]methyl]benzoyl]amino]ethoxy]ethoxy]ethoxy]ethyl]carbamate (55.8 mg, 0.084 mmol) and stirred for 2 hours before evaporation of solvent under reduces pressure. Redisolved in 500 mL of a 1:2 mixture of DIPEA and DMF and 3',6'-dihydroxy-6-isothiocyanato-spiro[isobenzofuran-3,9'-xanthene]-1-one hydrochloride (36 mg, 0.093 mmol) was added in one portion and stirred at RT for 2 hours protected from light. Solvents evaporated under reduced pressure and purified by reverse phase chromatography to yield 5-[2-[2-[2-[2-[[4-[[(2-amino-3-methyl-quinoline-6-carbonyl)-isobutyl-amino]methyl]benzoyl]amino]ethoxy]ethoxy]ethoxy]ethylcarbamothioylamino]-2-(3-hydroxy-6-oxo-xanthen-9-yl)benzoic acid 1 mg (1 %) with a 70 % purity by UV at 254 nm.

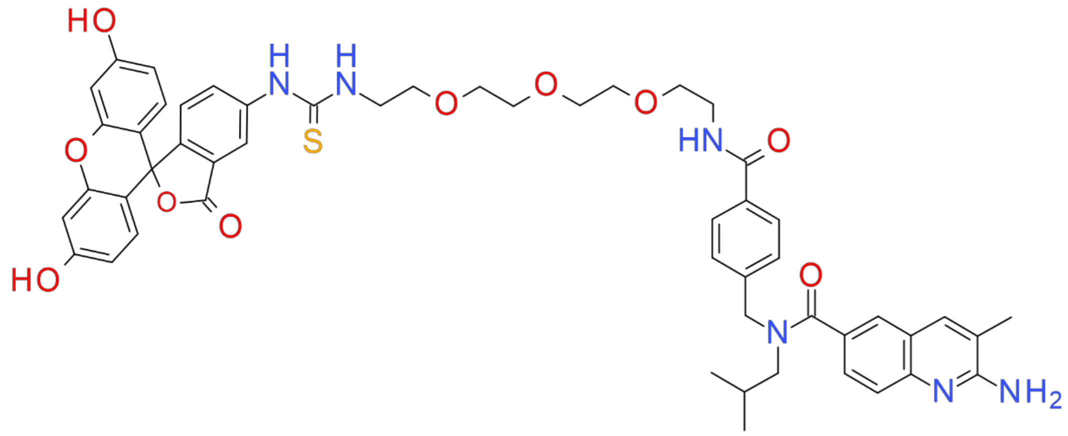

FIG S2. Chemical structure of the FITC-labelled derivative of AM-9934.

Synthesis of oligonucleotide-conjugates:

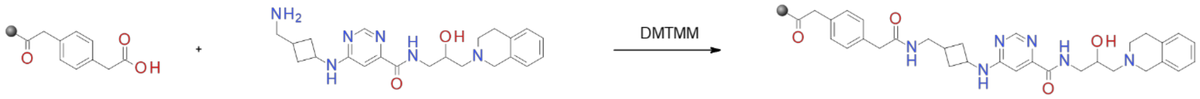

Synthesis of an oligonucleotide-conjugated EPZ015666-derivative (Grey dot denote oligonucleotide linkage site).

6-[[3-(aminomethyl)cyclobutyl]amino]-N-[3-(3,4-dihydro-1H-isoquinolin-2-yl)-2-hydroxy-propyl]pyrimidine-4-carboxamide was loaded onto an acid-functionalized oligonucleotide on DEAE Sephadex A-25 (GE Healthcare) in a 1 mL filter tube using the following protocol:

1. Wash immobilized oligo with 3 x 2vol DMF (200 µL) and drain
2. Add 32.5 µL 2% TEA in DMF (freshly prepared)
3. Add 12.5 µL of compound as 100 mM in DMSO
4. Add 7 µL 0.36 M DMT-MM in water
5. Incubate at 30°C for min. 10 min, 600 rpm
6. Drain
7. Repeat load (step 3-7)
8. Wash with 3 x 2vol DMF (200 µL) and drain
9. Wash with 5 x 2vol H_2O (200 µL) and drain

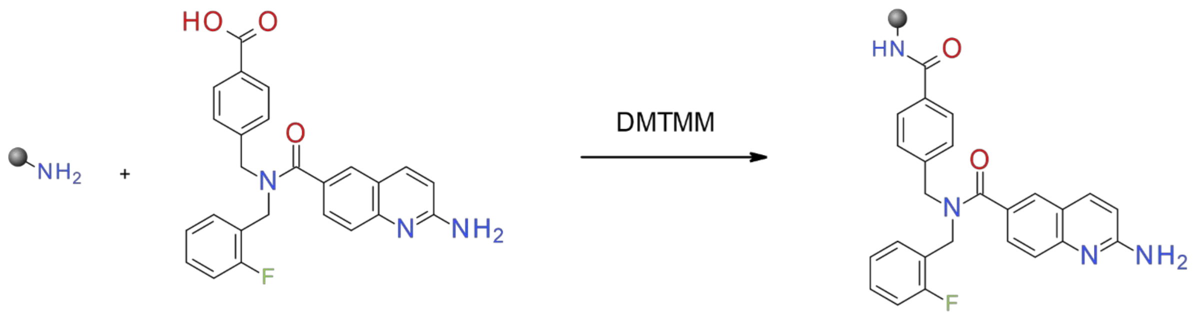

Synthesis of an oligonucleotide-conjugated AM-9959-derivative (Grey dot denote oligonucleotide linkage site).

4-[[(2-aminoquinoline-6-carbonyl)-[(2-fluorophenyl)methyl]amino]methyl]benzoic acid was loaded onto an amine-functionalized oligonucleotide 140using the following protocol:

1. Add to each well 5 µL 200 mM sodium phosphate buffer pH 8.0
2. Add 4 µL 100 mM BB in DMSO
3. Add 1 µL 360 mM DMT-MM in water (always use a new tube)
4. Spin down
5. Incubate at 30° C O/N in incubator
6. Add 140 µL H_2O to each well and incubate 10 min on plate shaker

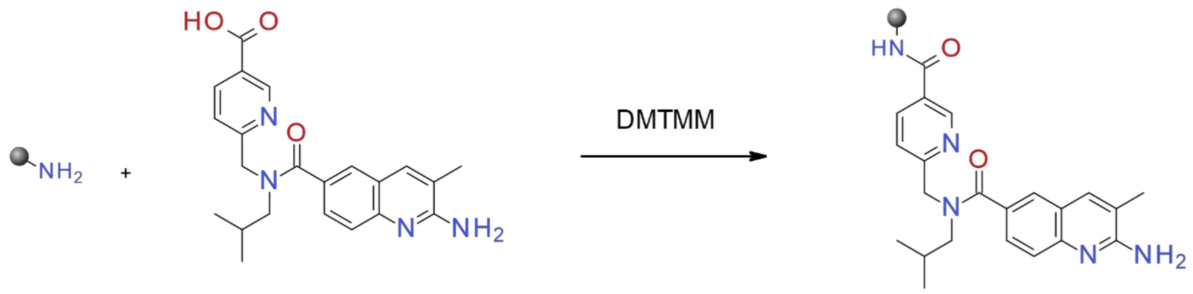

Synthesis of an oligonucleotide-conjugated AM-9934-derivative (Grey dot denote oligonucleotide linkage site).

6-[[(2-amino-3-methyl-quinoline-6-carbonyl)-isobutyl-amino]methyl]pyridine-3-carboxylic acid was loaded onto an amine-functionalized oligonucleotide using the following protocol:

1. Add to each well 5 µL 200 mM sodium phosphate buffer pH 8.0
2. Add 4 µL 100 mM BB in DMSO
3. Add 1 µL 360 mM DMT-MM in water (always use a new tube)
4. Spin down
5. Incubate at 30° C O/N in incubator

6. Add 140 µL H_2O to each well and incubate 10 min on plate shaker

Generalized protocol for immobilization of oligonucleotides (40 nmol) on DEAE

1. Add 100 µL of DEAE to a 1 mL filter tube
2. Drain
3. Wash with 2 x vol H_2O (200 µL)
4. Add “bottom” cap
5. Add oligo (40 nmol)
6. Incubate at 25°C for 15 min at 600rpm
7. Drain

Generalized protocol for elution of oligonucleotides with TEAB from DEAE filter tubes

1. Add 35 µl 2M TEAB pH 8.5 (for 10 nmol oligo) and incubate for 10 min 600 rpm
2. Spin at 1000 g for 1 min, collect the eluate in an eppendorf tube.
3. Repeat step 2-3
4. Flush tube with 70 µl H_2O
5. Combine the eluates from step 3 and 4 and purify using a pre-conditioned CentriPure N2 column washed with 2x200 µL of water.
6. Elute with 300 µL of water.

Biology

Fluorescence Polarization assay

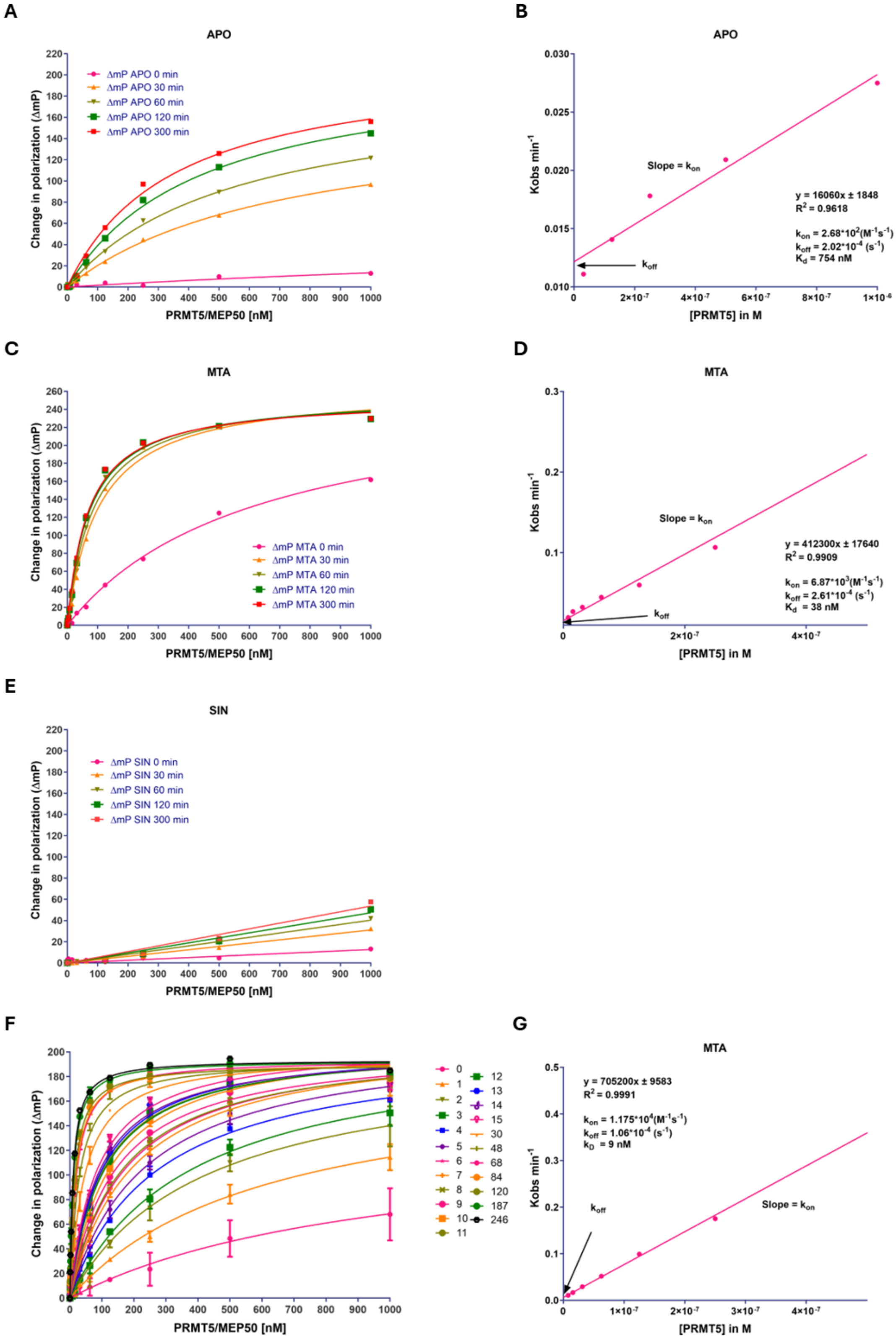

Figure S3. Fluorescence polarization assay demonstrating binding of FITC-labelled AM-9934-derivative to PRMT5 at the indicated time points (minutes) without (A) or with MTA (C) or SIN (E) in the presence of indicated concentrations of PRMT5:MEP50 complex. B,D. Lineweaver-Burk plots and calculated parameters (insets) of the data in A and C, respectively. No fit was possible for the data in E. F. Fluorescence polarization assay demonstrating binding of 1 nM of FITC-labelled AM-9934-derivative to PRMT5 at the indicated time points in minutes with MTA in the presence of indicated concentrations of PRMT5:MEP50 complex. G Lineweaver-Burk plot and calculated parameters (insets) of the data in F.

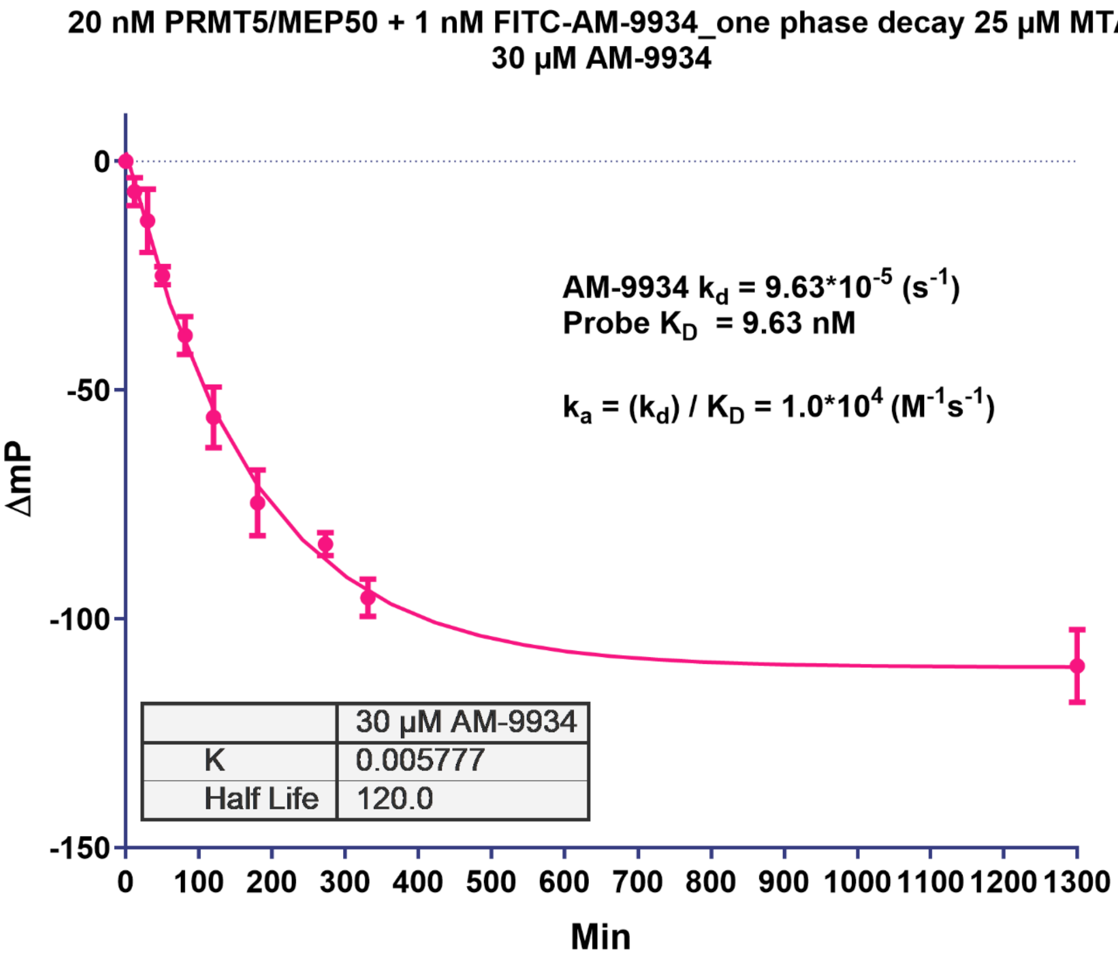

	30 µM AM-9934
K	0.005777
Half Life	120.0

Figure S4. Fluorescence polarization dissociation experiment after addition of excess of AM-9934 to pre-formed FITC-labelled AM-9934-derivative complex with PRMT5. Inset: Calculated parameters.

Human Bone Marrow Megakaryocyte Cell Viability Assay.

Human bone marrow CD34$^+$ progenitor cells from a normal healthy donor (#70002.4, Lot #2111403005, Donor ID CE0007310) and megakaryocyte expansion medium containing

SCF, IL-6, IL-9, and TPO were obtained from Stem Cell Technologies. Human bone marrow $CD34^+$ progenitor cells were cultured for four days in megakaryocyte expansion medium using the manufacturer's protocol (Stem Cell Technologies). Cells were seeded in 24-well plates and treated with DMSO, AM-9934 and EPZ015666 (0.014, 0.041, 0.123, 0.37, 1.11, 3.33, 10.0, 30.0 µM), LLY-283 (1.0 µM), paclitaxel (0.1 µM, cytotoxic control), or palbociclib (1.0 µM, cytostatic control). Cells were collected after 8 days (day 12 post-thaw). The megakaryocyte expansion medium with DMSO or compound was replenished on day 4 after treatment. Cells were collected and stained in a total of 100 µL of wash buffer (PBS, 0.5% BSA) with anti-hCD34 BV421 (562577, BD, mouse, 5 µL), and megakaryocyte markers anti-hCD41a FITC (555466, BD, mouse, 20 µL), and anti-hCD42b PE (555473, BD, mouse, 20 µL). The cells were also stained using matched isotype control antibodies. Cells were incubated on ice for 45 minutes and washed twice. Cells were resuspended in 200 µL of wash buffer plus 1 µL of LIVE/DEAD Fixable Near-IR Dead Cell Stain (L10119, Invitrogen) and incubated on ice for 30 minutes. Cells were washed twice in Wash/Perm buffer (554723, BD) and then fixed with Cytofix/Cytoperm buffer (554714, BD) and incubated overnight at 4°C. Cells were washed twice and passed through a blue-capped cell strainer tube (352235, Falcon) at a final volume of 800 µL. An equal number of counting beads (CountBright Plus Absolute Counting Beads, C36995, Invitrogen) were added to each sample tube and mixed by vortexing before data acquisition. Cells were analyzed with a BD LSRFortessa flow cytometer running FACSDiva software. A stop gate was set at a 10,000-bead cutoff value for each sample. Post-acquisition analysis was performed using FSC Express software. The flow cytometry gating scheme is as follows: 1) viable cell primary size gate, 2) $CD34^+$ cell population progenitor secondary gate, and 3) $CD41a^+$ and $CD42b^+$ cell population megakaryocyte secondary gate (FIG. S6). A relative viable cell count was determined for the $CD41a^+$ and $CD42b^+$ cell population. The $CD41a^+$ and $CD42b^+$ cell count POC values were computed using the formula ((compound-treated count) ÷ (DMSO-treated count) × 100). Count data were graphed as a concentration-response profile with the corresponding count EC_{50} values. The curve fitting (four-parameter non-linear regression equation) was performed using GraphPad Prism version 10.2.1 software (GraphPad Software).

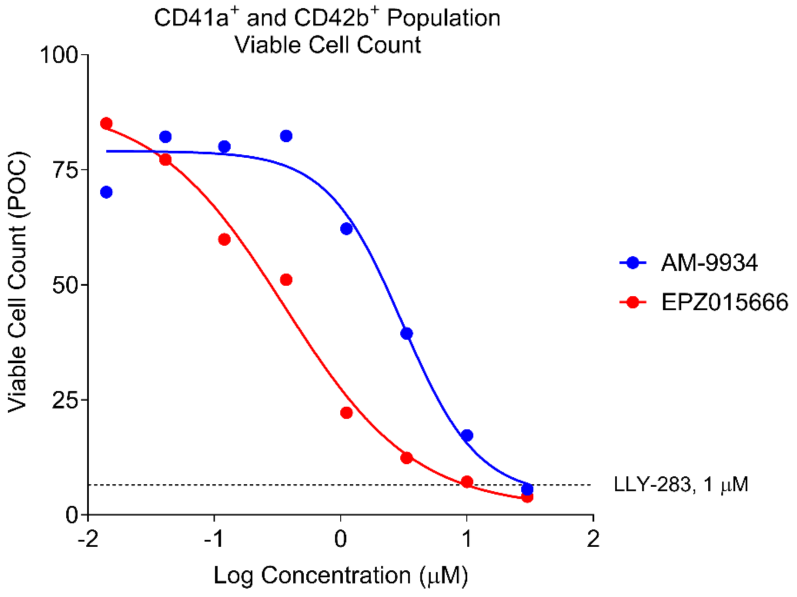

Figure S5. PRMT5 inhibitor effects on the viability of human bone marrow megakaryocyte cells from a normal healthy donor. Cell viability analysis of human bone marrow megakaryocyte cells (CD41a^{+} and CD42b^{+}) performed after 8 days of treatment with DMSO, AM-9934 and EPZ015666 (0.014, 0.041, 0.123, 0.37, 1.11, 3.33, 10.0, 30.0 μM), or LLY-283 (1.0 μM). Concentration-response profiles presented as a viable cell count relative to the percentage of DMSO control (POC), with AM-9934 and EPZ015666 IC_{50} values of 3.1 μM and 0.36 μM, respectively. Dashed line indicates the viability effects of PRMT5 inhibitor LLY-283. The flow cytometry gating scheme is shown in FIG. S6

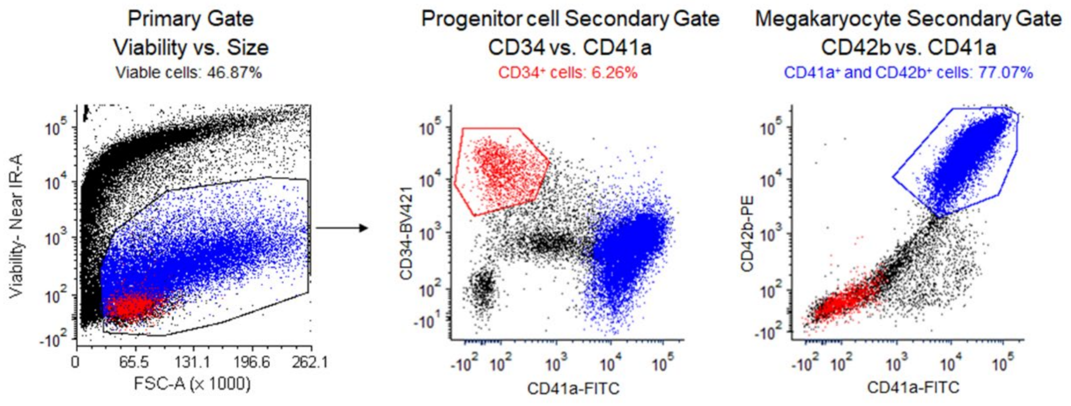

Figure S6. Flow cytometry gating scheme for human bone marrow CD34^{+} cells cultured in megakaryocyte expansion medium and treated with DMSO for 8 days. Cells stained to detect the viable CD34^{+} progenitor cell population (red) and CD41a^{+} and CD42b^{+} megakaryocyte cell population (blue).

X-RAY Crystallography

Table S1 | Data collection and refinement statistics

	PRMT5-MEP50-MTA-AM-9934
**Data collection**	
Space group	I222
Cell dimensions	
a, *b*, *c* (Å)	104.26, 138.38, 178.43
α, β, γ (°)	90, 90, 90
Resolution (Å)	41.4 - 3.1 (3.211 - 3.1)
Rmerge	0.098(0.507)
$I / \sigma I$	19.2(5.0)
Completeness (%)	99.9(100)
Multiplicity	12.9(12.9)
$CC_{1/2}$	0.998/0.984
**Refinement**	
Resolution (Å)	(3.211 - 3.1)
No. reflections	23578
R_{work} / R_{free}	0.21/0.27
No. atoms	
Protein	7345
Ligand/ion	50
Water	23
B-factors	
Protein	113.54
Ligand/ion	86.25
Water	75.09
R.m.s. deviations	
Bond lengths (Å)	0.002
Bond angles (°)	0.46

*Values in parentheses are for highest-resolution shell.
